# Comparison of four techniques for spine stereotactic body radiotherapy: Dosimetric and efficiency analysis

**DOI:** 10.1002/acm2.12271

**Published:** 2018-02-07

**Authors:** Saif Aljabab, Balamurugan Vellayappan, Eric Vandervoort, Jamie Bahm, Robert Zohr, John Sinclair, Jean‐Michel Caudrelier, Janos Szanto, Shawn Malone

**Affiliations:** ^1^ Radiation Medicine Program The Ottawa Hospital Cancer Centre Ottawa ON Canada; ^2^ Ottawa Hospital Research Institute Ottawa ON Canada; ^3^ Division of Radiation Oncology Department of Radiology University of Ottawa Ottawa ON Canada; ^4^ Division of Neurosurgery University of Ottawa Ottawa ON Canada; ^5^ Division of Radiation Oncology National University Cancer Institute Singapore

**Keywords:** conformity index, cyberknife, dosimetric evaluation, dynamic wedges, spine SBRT, tomotherapy, VMAT

## Abstract

**Purpose:**

The aim of this study is to compare the dosimetric differences between four techniques for spine stereotactic body radiotherapy (SBRT): CyberKnife (CK), volumetric modulated arc therapy (VMAT), and helical tomotherapy (HT) with dynamic jaws (HT‐D) and fixed jaws (HT‐F).

**Materials/methods:**

Data from 10 patients were utilized. All patients were planned for 24 Gy in two fractions, with the primary objectives being: (a) restricting the maximum dose to the cord to ≤ 17 Gy and/or cauda equina to ≤ 20 Gy, and (b) to maximize the clinical target volume (CTV) to receive the prescribed dose. Treatment plans were generated by separate dosimetrists and then compared using velocity AI. Parameters of comparison include target volume coverage, conformity index (CI), gradient index (GI), homogeneity index (HI), treatment time (TT) per fraction, and monitor units (MU) per fraction.

**Results:**

PTV D2 and D5 were significantly higher for CK compared to VMAT, HT‐F, and HT‐D (*P* < 0.001). The average volume of CTV receiving the prescription dose (CTV D95) was significantly less for VMAT compared to CK, HT‐F and HT‐D (*P* = 0.036). CI improved for CK (0.69), HT‐F (0.66), and HT‐D (0.67) compared to VMAT (0.52) (*P* = 0.013). CK (41.86) had the largest HI compared to VMAT (26.99), HT‐F (20.69), and HT‐D (21.17) (*P* < 0.001). GI was significantly less for CK (3.96) compared to VMAT (6.76) (*P* = 0.001). Likewise, CK (62.4 min, 14059 MU) had the longest treatment time and MU per fraction compared to VMAT (8.5 min, 9764 MU), HT‐F (13 min, 10822 MU), and HT‐D (13.5 min, 11418 MU) (*P* < 0.001).

**Conclusion:**

Both CK and HT plans achieved conformal target coverage while respecting cord tolerance. Dose heterogeneity was significantly larger in CK. VMAT required the least treatment time and MU output, but had the least steep GI, CI, and target coverage.

## 1 BACKGROUND

The clinical burden of osseous metastatic disease has increased due to patients surviving longer from advances in systemic therapy. Up to 40% of these patients have spinal metastasis.^1^ Various techniques, including dose‐escalated radiotherapy, have been utilized to improve local control and symptom palliation.^2^, ^3^, ^4^, ^5^ Safe dose escalation using stereotactic body radiotherapy (SBRT) translates into durable pain relief, decompression of epidural extensions and local control rates of 70%–90%.^6^, ^7^, ^8^, ^9^, ^10^, ^11^, ^12^

Horse‐shoe shaped target volumes, which are common in spine SBRT, increase the complexity of such techniques, affecting target volume coverage. Moreover, the proximity of this concave target to the spinal cord mandates a highly conformal radiotherapy plan with sharp dose gradients.^13^ The expansions of a clinical target volume (CTV) to a planning target volume (PTV) and of organs at risk (OAR) to a planning organ at risk volume (PRV) increases the likelihood of overlap between targets and critical structures. This can potentially increase plan complexity and technical demand on the delivery system.^14^ Aside from radiation myelopathy, treatment‐related vertebral compression fracture (VCF) is a known complication from SBRT.^15^, ^16^ It remains possible that the dose heterogeneity (i.e., hotspots) within the vertebral body contributes to the risk of VCF. With multiple modalities of treatment available, it remains unclear if a particular technique offers a good compromise between target volume coverage, sparing of critical structures and treatment time.

The CyberKnife (CK) unit (Accuray, Sunnyvale, CA, USA) is a frameless nonisocentric 6 MV linear accelerator (LINAC) mounted on a computer‐controlled precise six‐axis manipulator (robotic arm). It uses a pair of planar orthogonal kilovoltage imaging systems to monitor the patient position and orientation throughout their treatment. It has been extensively described elsewhere.^17^ CK is a commonly used modality to treat spine‐based metastasis due to its highly conformal dose distributions, steep gradient, and near real‐time image‐guidance system.^18^, ^19^, ^20^, ^21^, ^22^

Volumetric modulated arc therapy (VMAT) is a rotational intensity modulated radiotherapy (IMRT) treatment that is more widely available and increasingly common for spine SBRT. This planning study utilized the VMAT capabilities of a Synergy LINAC (Elekta AB, Stockholm, Sweden) equipped with the Agility multileaf collimator (MLC). This MLC has a leaf width of 5 mm as projected at the isocenter 100 cm from the source, a maximum MLC leaf speed of 65 mm/s, and nearly continuously variable dose rate (256 different bins with a nominal maximum of 9.25 monitor units/s), as well as variable gantry speed (maximum 6°/s). The gantry rotates, up to 360°, around the patient during radiation delivery.^23^ Studies comparing VMAT to fixed IMRT revealed overall equivalence in conformality and homogeneity but with a marked decrease in treatment time particularly when 2 passes per arc where employed rather than just a single rotation.^24^, ^25^, ^26^

Helical tomotherapy (HT) is a technique which utilizes a compact 6 MV LINAC mounted on a rotating slip ring gantry, similar to that used for computed tomography (CT), with 64 multileaf binary MLC which projects to 0.625 cm width at an isocenter 85 cm away from the source. HT has been used mainly with fixed fan‐beam jaws (HT‐F) with widths of 1, 2.5, and 5 cm lengths available for planning. More recently, dynamic jaws have been implemented (HT‐D) to help reduce beam penumbra at the superior and inferior portions of the treated volume. This system can provide highly homogeneous and conformal dose distributions through a helical photon delivery system at a constant speed with a continuously moving couch combined with rapid binary MLC beam modulation.^24^, ^27^, ^28^, ^29^

There have been other dosimetric studies comparing spine SBRT techniques. The study by Ma et al. compared CK, intensity modulated proton therapy, fixed‐field IMRT, and VMAT for one, two, and three thoracic vertebral bodies.^2^ They reported that all modalities studied were able to achieve acceptable planning constraints. However, CK had the highest degree of target dose inhomogeneity. This study did not compare HT to these modalities nor report on efficiency of treatment delivery. Efficiency of treatment delivery is particularly important for spine SBRT as majority of the patients are in pain and intrafraction motion increases with prolonged treatments.

Another publication by Yang et al., compared CK, RapidArc (Varian), IMRT, and HT‐F.^30^ They restricted the study population to anterior thoracic vertebrae lesions (body ± pedicle) and allowed a large variation in dose prescription (between 33 Gy in three fractions and 40 Gy in five fractions). This study also looked at treatment efficiency as one of the endpoints. Like the previous study, CK had the most dose heterogenenity but had the longest treatment time. IMRT had poorer coverage compared to CK, RA, and HT, for both body‐type lesions and body with pedicle lesions. Two other publications have reported that target volume coverage is inferior with fixed beam IMRT compared to VMAT.^25^, ^31^

In view of the above reports, we chose not to compare fixed‐field IMRT with the other techniques. Our initial experience suggested that HT‐D may have better dose conformity than HT‐F. Moreover, previous reports have suggested that treatment time can be reduced between 40% and 50%^32^ compared to HT‐F. However, it has yet to be formally compared with other techniques in the setting of spine SBRT.

Our objective was to conduct a dosimetric and treatment efficiency analysis between the above modalities. In particular, we preferred to use a uniform prescribed dose and planning priorities while making this comparison. Our findings may help to guide cancer centers, with more than one of these technologies, on resource allocation.

## 2 MATERIALS AND METHODS

### 2.A Study design

This study is a single center retrospective dosimetric study of 10 patients previously treated with spine SBRT with CK. It was approved by the local institutional review board. All cases were due to metastatic spine disease, without any restrictions on tumor histology, and included a single vertebral level from C1–L2.

### 2.B Imaging and contouring

Treatment planning CT scans (1.0 mm axial slices) were obtained for each patient in supine position with an evacuated vacuum cushion for lesions below T3, and in a patient‐specific thermoplastic shell for lesions T3 and above. CT images were co‐registered with a recent T1 axial post gadolinium and T2 axial magnetic resonance imaging (MRI).

Targets were contoured based on the International Spine Radiosurgery Consortium Consensus Guidelines.^14^ Targets were drawn by an experienced radiation oncologist, and standardized for all cases. The CTV for soft tissue extension was a gross target volume (GTV) with a 2 mm margin as well as the adjacent elements of spine anatomy, as per consortium guidelines. This was expanded uniformly by 1.5 mm to form the PTV. Spinal cord and cauda were contoured, based on the T2 MRI, at least two vertebrae levels beyond the target both superiorly and inferiorly. A PRV was contoured at 1.5 mm from the cord and cauda. Other OAR, including esophagus, cardiovascular structures, intestines, kidneys, were contoured depending on the location of PTV.

### 2.C Planning objectives

Prescribed treatment dose was 24 Gy in two fractions with a biological effective dose ($BED_{10}$) of 52.8 Gy and $BED_{3}$ of 120 Gy. The planning goals were to maximize the volume of the CTV received 100% of the prescribed dose, while giving priority to the PRV constraints for the spinal cord, cauda equine, and brainstem. This regimen is the standard palliative spine SBRT schedule used at our center. A dose constraint of D_0.25 cc_ ≤ 17 Gy in two fractions was used for both the cord and brain stem PRVs; for the cauda equina PRV, the dose constraint used was D_0.25 cc_ ≤20 Gy in two fractions. The D_0.25 cc_ parameter was picked as opposed to D_max_ to better compare between the modalities. Each planning system has a different sized calculation of approximately 1 × 1 × 1 mm^3^ for CK (0.001 cc), 2 × 2 × 1 mm^3^ for HT (0.004 cc), and 2 × 2 × 2 for VMAT (0.008 cc). This translates the D_max_ value differently in each system. As per ICRU 83,^33^ this is a near max dose that can be used.

CT datasets with contours were exported to the respective modality software platform. Different maximum dose constraints to the PTV were allowed for the different modalities since the dose heterogeneity achievable depends on the delivery technique and dose calculation method used. Treatment plans were generated by separate dosimetrists on each modality and evaluated by a radiation oncologist before exporting it to velocity AI for rescaling and dosimetric evaluation. Rescaling was required in 8 out of 40 plans (VMAT = 1, CK = 3, HT‐D = 3, HT‐F = 1) in order to meet the specified spinal cord or cauda constraints as reported in the VelocityAI software. This is necessary to account for small differences in how the different planning systems and VelocityAI account for intersections between voxels and contours. The rescaling factors were generally small (average scaling factor = 1.04 with a standard deviation of ± 0.06) and are not expected to impact the deliverability of these plans.

### 2.D CyberKnife

CK plans were generated using sequential optimization in MultiPlan^®^ Treatment Planning Software version 4.6. A total of two to three fixed circular collimators were used for each plan, with an initial maximum number of monitor units of 30,000 and a goal of less than 70 min total estimated treatment time. The primary planning goal was to achieve the maximum PTV coverage, allowing a maximum dose of 32 Gy to the PTV, while maintaining the dose limiting OAR constraint for the cord and/or cauda equina PRV. Secondary goals were to minimize the dose to three conformal ring structures at 5, 15, and 30 mm from the PTV. All the final dose calculations were done using the Monte‐Carlo method with 1% uncertainty and a fine calculation grid with a voxel size the same as the CT image approximately equal to 0.1 cm × 0.1 cm × 0.1 cm.

### 2.E Volumetric modulated arc therapy

VMAT plans were generated using the Monaco^®^ Treatment Planning Software version 3.2. For all plans, one to three arc segments were used with a maximum of two passes per arc. Low fluence smoothing was used with 150 control points per arc and a minimum segment width of 1 cm. Typically, plans were optimized in constrained mode with target penalties and quadratic overdose cost functions used to maximize PTV coverage and control high doses in the target, respectively, while quadratic overdose and serial cost functions were used for PRVs and OARs. A maximum dose of 28 Gy was allowed to the PTV, while maintaining the dose limiting OAR constraints for the PRVs. The Monte‐Carlo method was used for dose‐to‐medium calculation with 3% uncertainty per control point and a calculation voxel size of 0.2 cm × 0.2 cm × 0.2 cm.

### 2.F Helical tomotherapy—fixed and dynamic jaws

Two HT plans were optimized using the TomoTherapy Planning Station version 5.0. Using a 1 cm beam width would slightly improve the dose distribution but dramatically increase treatment time (two‐ to threefolds) which would make it less practical. For that reason, a 2.5 cm beam width was selected to better compare the HT‐D and HT‐F outcomes. A pitch of 0.287 and modulation factor ranging from 2.1 to 2.7 was used for optimization. During planning, the user can interactively change the maximum dose and dose volume constraint penalties and their respective weights in the optimization cost function. The primary objective during planning was to achieve the highest volume of PTV to be covered by the prescription dose while maintaining a maximum dose of 26.4 Gy to the PTV and respecting the dose limiting OAR constraints for the PRVs. The total dose for each plan was divided into four fractions, as the maximum gantry period of 60 s was exceeded when divided into two fractions. Therefore, if these plans were to be delivered, two calculated fractions would have to be delivered in one treatment session to deliver the prescribed dose per fraction. The collapsed cone method is used by this planning system for dose calculation with a fine calculation grid size approximately equal to 0.2 cm × 0.2 cm × 0.1 cm.

### 2.G Dosimetric evaluation

For the PTV coverage assessment, dose volume histogram “shoulder” metrics (near minimum), D_98%_ and D_95%_, dose which is received by 98% and 95% of the PTV volume, respectively, and “tail” metrics (near maximum) D_5%_ and D_2%_ was compared. The target coverage of the CTV (D_95%_) was assessed. In addition, the treatment time and the total number of monitor units (MU) per fraction were compared. As the plan priority was not to exceed D_0.25 cc_, the near minimum dose to 0.25 cc volume of the cord and cauda equina PRV, no difference was expected among the techniques; hence, a formal comparison was not be made.

Homogeneity index (HI) quantifies dose homogeneity within the target volume. Lower values indicate superior homogeneity with an index of 0 representing the most homogeneous plan possible.^26^, ^34^ HI was calculated as below:

$$HI_{2/98}=\frac{D_{2\%}-D_{98\%}}{D_{\mathrm{prescription}}}\times 100\%$$

*D*_*prescription*_ is the prescription dose.

Conformity Index (CI) is a metric which evaluates how well the prescribed isodose line envelopes the exact shape of the target volume. We used a global CI equation:

$$CI\left(\mathrm{PTVGlobal}\right)=\frac{TV_{\mathrm{RI}}}{\mathrm{TV}}\times \frac{TV_{\mathrm{RI}}}{V_{\mathrm{RI}}}$$

$V{}_{\mathrm{RI}}$ = volume inside the reference isodose contour in cc, TV = target volume in cc and $TV_{\mathrm{RI}}$ = target volume covered by the reference isodose contour in cc. Note that, for this definition, a CI < 1 can occur in cases of both over and under coverage. In a perfect plan, the CI would be equal to 1. The reference isodose used was the prescription dose of 24 Gy.^35^, ^36^

Gradient index (GI) is a volumetric measure of how rapidly the dose falls off from the prescription isodose line. For this study, we used:

$$GI=\frac{V_{12\;Gy}}{V_{24\;Gy}}$$

We used the ratio of the total volume receiving half (12 Gy) and the full prescribed dose (24 Gy). A GI closer to 1 means adjacent OAR or other normal tissue will receive less dose due to the dose sharper gradient.^18^, ^30^

### 2.F Statistical analysis

Statistical analysis was performed with SPSS (v21, Chicago, USA). Data are expressed as mean ± standard deviations. The dosimetric characteristics of the four techniques were analyzed using the one‐way ANOVA. Post‐hoc tests with the Bonferroni's method for multiple comparisons were used to further analyze the differences. Values of *P* < 0.05 were considered statistically significant for one‐way ANOVA. Values of *P* < 0.01 were considered statistically significant for multiple comparisons.

## 3 RESULTS

### 3.A Dataset

A total of 10 patients with 10 lesions who have undergone spine SBRT using the CK unit were identified from 2010 to 2014. A total of two cervical, four thoracic, and four lumbar spine vertebral bodies were included. Eight cases involved the vertebral body with and without pedicles (anterior lesions) and two cases involved the posterior spinous process alone (posterior lesions). GTV volumes ranged from 1.5 to 62.8 cc and PTV volumes ranged from 16.7 to 95.7 cc. Patient and tumor characteristic details are outline in (Table 1).

**Table 1:** Patient and tumor characteristics

Case No.	Age	Gender	Primary cancer diagnosis	Spine level	Vertebral component	GTV target volumes in (cc)	PTV target volumes in (cc)
1	56	F	Melanoma	T6	Anterior	1.5	22.3
2	52	F	Breast	T5	Anterior	4.4	16.7
3	82	M	Lung	C2	Anterior	7.6	18.6
4	81	M	Prostate	C1	Anterior	14.4	20.7
5	67	M	Prostate	T9	Anterior	14.6	33.9
6	65	F	Renal	L2	Posterior	20.6	57.9
7	57	F	Breast	L2	Anterior	25.6	72.2
8	66	F	Breast	L1	Anterior	27.6	78
9	52	M	Hemangioma	L2	Anterior	55.1	67.6
10	78	F	Lung	T6	Posterior	62.8	95.7

### 3.B Dosimetric analysis

**Figure 1:**
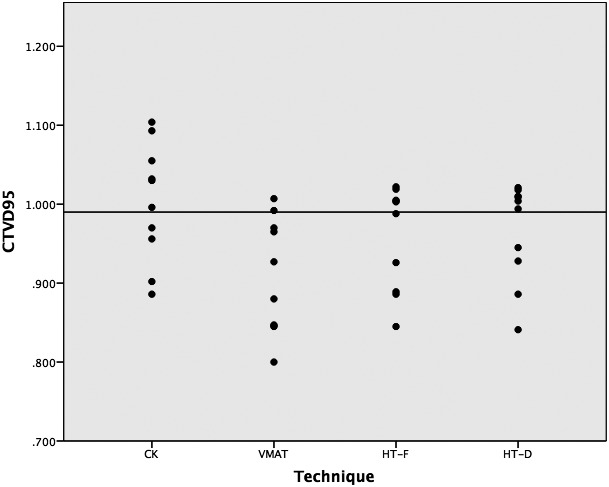
Modality coverage of CTV. Dot = CTV% coverage per modality; line = CTV 95% coverage.

**Figure 2:**
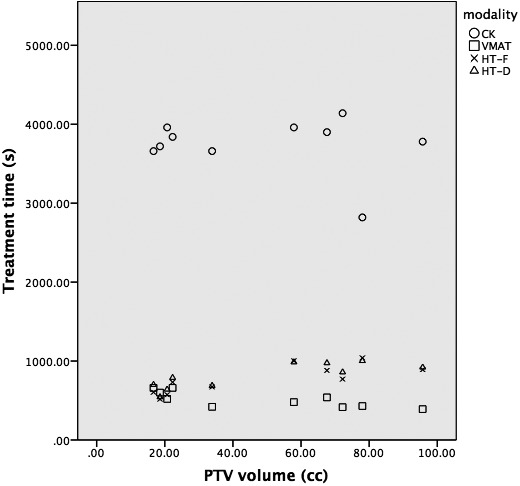
Treatment time comparison. Circle = CK; Square = VMAT; cross = HT‐F; triangle = HT‐D.

**Figure 3:**
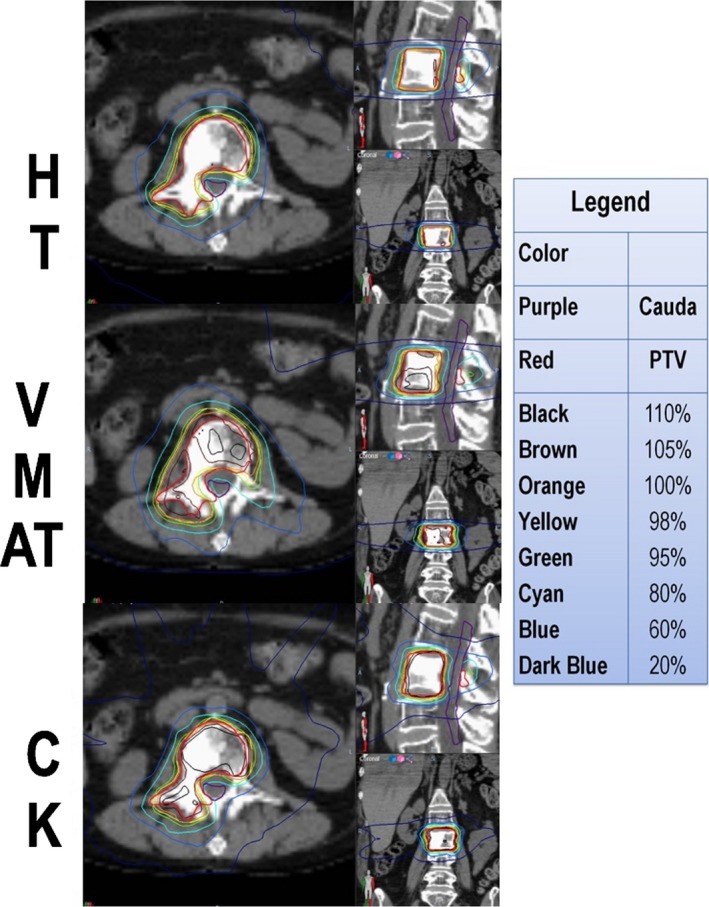
Case 4 dose distribution comparison. Purple = cauda equina; red = PTV; black = 110% isodose line; brown = 105%; orange = 100%; yellow = 98%; green = 95%; cyan = 80%; blue = 60%; dark blue = 20%.

As expected by study design, there was no significant difference between the four techniques in the maximum dose to the cord or cauda equine. Other dosimetric comparisons are outlined in (Table 2). The PTV D_98%_ and D_95%_ were not significantly different between the techniques. The CTV D_95%_ = ≥ 99% criterion coverage was satisfied with six cases in CK, two in VMAT, five in HT‐F, and six in HT‐D (Figure 1). The maximum dose to the PTV (D2, D5) was significantly higher with CK (*P* < 0.001). The CI was improved for CK and HT compared to VMAT; however, it was not different between CK and HT. The GI of CK was significantly better than VMAT, but not for the other techniques. Likewise, the HI, treatment time, and MU were highest for CK in comparison to VMAT and HT (Table 3). The treatment time was consistently higher for CK than VMAT and HT across the range of PTV volumes (Figure 2). Sample contours and dose distribution for Case 4 are shown in Figure 3.

**Table 2:** Dosimetric results of the modalities used

	CK	VMAT	HT‐F	HT‐D	F	*P*‐value
PTV D98	0.87	0.83	0.86	0.86	0.55	0.65
Mean (SD)	(0.08)	(0.08)	(0.08)	(0.08)		
PTV D95	0.95	0.88	0.92	0.93	1.66	0.19
Mean (SD)	(0.07)	(0.08)	(0.07)	(0.07)		
PTV D5	1.27	1.09	1.07	1.07	77.06	<0.001*
Mean (SD)	(0.06)	(0.02)	(0.02)	(0.03)		
PTV D2	1.29	1.10	1.07	1.07	86.81	<0.001*
Mean (SD)	(0.06)	(0.02)	(0.02)	(0.03)		
CTV D95	1.00	0.91	0.96	0.97	3.15	0.036
Mean (SD)	(0.09)	(0.07)	(0.07)	(0.06)		
CI	0.69	0.52	0.66	0.67	4.09	**0.013**
Mean (SD)	(0.10)	(0.15)	(0.12)	(0.11)		
GI	3.96	6.76	5.84	4.86	4.47	0.009*
Mean (SD)	(0.83)	(3.23)	(1.12)	(0.89)		
HI	41.86	26.99	20.69	21.17	13.17	<0.001*
Mean (SD)	(7.27)	(8.85)	(8.80)	(9.37)		
TT	3744	512	769	810	473.5	<0.001*
Mean (SD)	(358)	(101)	(181)	(162)		
MU	14059	9763	10822	11418	6.47	0.001*
Mean (SD)	(1719)	(2317)	(2622)	(2340)		
PRV Cord	16.18	16.38	16.47	16.25	0.11	0.96
Mean (SD)	(1.33)	(1.09)	(0.55)	(0.77)		
PRV Cauda	19.91	19.70	19.75	19.70	0.59	0.63
Mean (SD)	(0.06)	(0.34)	(0.26)	(0.26)		

*indicates statistically significant P value.

CK = CyberKnife; VMAT = volumetric modulated arc therapy; HT‐D = helical tomotherapy with dynamic jaws; HT‐F = helical tomotherapy with fixed jaws.

**Table 3:** Dosimetric comparison of the four modalities

	*P*‐values					
	CK vs VMAT	CK vs HT‐F	CK vs HT‐D	VMAT vs HT‐F	VMAT vs HT‐D	HT‐F vs HT‐D
PTV D98	0.23	0.77	0.78	0.37	0.36	0.99
PTV D95	0.04	0.41	0.45	0.18	0.16	0.95
PTV D5	**<0.001** *	**<0.001** *	**<0.001** *	0.18	0.23	0.89
PTV D2	**<0.001** *	**<0.001** *	**<0.001** *	0.067	0.13	0.74
CTV D95	**0.004** *	0.17	0.24	0.11	0.07	0.83
CI	**0.004** *	0.58	0.79	0.016	**0.007** *	0.05
GI	**0.001** *	0.026	0.28	0.27	0.025	0.23
HI	**<0.001** *	**<0.001** *	**<0.001** *	0.11	0.14	0.90
TT	**<0.001** *	**<0.001** *	**<0.001** *	0.014	**0.005** *	0.68
MU	**<0.001** *	**0.003** *	0.014	0.31	0.11	0.56

*indicates statistically significant P value.

## 4 DISCUSSION

The utilization of SBRT techniques to treat spinal metastasis is increasing exponentially. As more techniques become available, it becomes important to choose the optimal technique for such cases. Factors to consider include target volume coverage, sparing of critical structures, and treatment time. This study conducted a dosimetric and treatment efficiency analysis of four modern techniques for spine SBRT.

Our study included a wide range of tumor sizes, vertebral levels, and included both anterior and posterior tumors. Targets were standardized and followed recommendations based on the International Spine Radiosurgery Consortium Consensus Guidelines. Consequently, CTV and PTV contours increased the final target volume by two‐ to fivefolds. The dose fractionation used was 24 Gy in two fractions. All treatment plans were normalized to meet the prespecified strict cord and/or cauda equina constraints of D_0.25 cc_ < 17 Gy. Available data from the literature reports the risk of radiation‐induced myelopathy with these constraints to be less than 1%.^13^, ^37^ We used 0.25 cc as opposed to 0.1 cc for cord constraints to account for voxel resolution inaccuracies and differences between the three software platforms.

Our findings suggest that all modalities were capable of generating acceptable treatment plans. CK and HT provided superior target coverage and sharpest dose gradients. However, the HI, treatment time, and MU were significantly higher with CK. There were no significant differences between HT‐F and HT‐D, including the treatment time. This was contradictory to previous reports^32^ and may be due to the small PTV volumes seen with spine SBRT. VMAT although having inferior target volume coverage, CI and GI had the shortest treatment times and required the least MU. CK consistently had the longest treatment times and MU, compared to HT and VMAT, across the range of PTV volumes.

Due to the nature of beam delivery on the CK unit, using many nonisocentric, overlapping circular radiation fields delivered from multiple noncoplanar directions, it is well recognized that dose within the PTV can be very heterogeneous for this modality, as high as 150% of the prescribed dose. It remains unclear how such heterogeneity impacts tumor control probability and normal‐tissue complication probability. Potentially, a higher maximum dose may lead to improved tumor control or contribute to toxicities such as vertebral compression fracture due to demineralization of the normal bone structure.^15^

It is also important to note that CK uses near real‐time tumor tracking, and may potentially require a smaller PTV margin, leading to a smaller treatment volume. Moreover, because of the real‐time tracking, intrafraction movement is less of a concern even though treatment times may be longer than other modalities. Another consideration to note is that HT must segment its dose delivery as it has a 6 Gy per fraction limit. In our study, for each 12 Gy fraction, there are two segments. To minimize risk of intrafraction motion, a midfraction MVCT is used at some HT centers, and this can contribute to increased treatment time and extra imaging dose to the patient.

We acknowledge some limitations of our study, besides the retrospective nature. The dosimetric nature of the study may have underestimated setup errors across the techniques as the patients were only treated on the CK unit. Second, although we did use a single platform to compare the treatment plans for all four modalities, the dose calculation differences between the different implementations of the Monte‐Carlo method and between collapsed cone calculation techniques, as well as dose grid size difference, and differences in the optimization algorithms are specific to each treatment planning platform prior to exporting the dose data. Third, the small sample size of 10 patients which has been historically used in dosimetric studies can affect statistical power calculations. Thus, the statistical results of such small studies should be interpreted with caution.

Future studies should consider comparing such techniques on the same treatment planning software and calculation algorithm. This would reduce the variability introduced by the above. Lastly, the impact of dose distribution on tumor control rates, palliative benefits, and rates of vertebral fracture are still yet unknown and prospective phase II/III trials (such as RTOG 0613, NCIC SC24) may provide valuable information.

## 5 CONCLUSION

We compared four techniques used for spine SBRT on 10 lesions, with the treatment plan being normalized to meet cord and cauda equina constraints. CK and HT had adequate coverage of the target volume, although CK had a higher PTV maximum dose and HI. Although VMAT had the lowest target volume coverage and CI, it had the shortest treatment time. HT‐F provided good balance between target volume coverage, treatment time and conformity. We did not find any perceivable benefits with the use of HT‐D over HT‐F. Centers with more than one of these modalities may find our results helpful in choosing one technique over the other. Improvements in technology, such as higher dose rate and improved treatment planning algorithm, may narrow the gap between different modalities.

## CONFLICT OF INTEREST

None.
